# Assessment of Mixed Sward Using Context Sensitive Convolutional Neural Networks

**DOI:** 10.3389/fpls.2020.00159

**Published:** 2020-02-27

**Authors:** Christopher J. Bateman, Jaco Fourie, Jeffrey Hsiao, Kenji Irie, Angus Heslop, Anthony Hilditch, Michael Hagedorn, Bruce Jessep, Steve Gebbie, Kioumars Ghamkhar

**Affiliations:** ^1^ Lincoln Agritech Limited, Lincoln University, Lincoln, New Zealand; ^2^ Red Fern, Solutions Limited, Christchurch, New Zealand; ^3^ Development Engineering, Lincoln Research Centre, AgResearch, Lincoln, New Zealand; ^4^ Forage, Science, Grasslands Research Centre, AgResearch, Palmerston North, New Zealand

**Keywords:** forage yield, clover, ryegrass, biomass, semantic segmentation, deep learning

## Abstract

Breeding higher yielding forage species is limited by current manual harvesting and visual scoring techniques used for measuring or estimation of biomass. Automation and remote sensing for high throughput phenotyping has been used in recent years as a viable solution to this bottleneck. Here, we focus on using RGB imaging and deep learning for white clover (*Trifolium repens* L.) and perennial ryegrass (*Lolium perenne* L.) yield estimation in a mixed sward. We present a new convolutional neural network (CNN) architecture designed for semantic segmentation of dense pasture and canopies with high occlusion to which we have named the local context network (LC-Net). On our testing data set we obtain a mean accuracy of 95.4% and a mean intersection over union of 81.3%, outperforming other methods we have found in the literature for segmenting clover from ryegrass. Comparing the clover/vegetation fraction for visual coverage and harvested dry-matter however showed little improvement from the segmentation accuracy gains. Further gains in biomass estimation accuracy may be achievable through combining RGB with complimentary information such as volumetric data from other sensors, which will form the basis of our future work.

## Introduction

The increase in demand for meat and dairy over the last few decades has led to an intensification of forage based farming. Breeding for the improvement of forage yield and nutrient composition of grassland forage species adds value to these industries (Smith and Spangenberg, 2014; Capstaff and Miller, 2018; Gebremedhin et al., 2019). The length of time required to develop stable new forage cultivars can however take up to 10–15 years (Lee et al., 2012). One of the bottlenecks in this process is that growth rates and yield measurements for these forage species are generally done by visual scoring and/or manual harvesting. Both of these practices require a considerable amount of time and labour to perform, which in turn limits the size of breeding trials (Araus et al., 2018; Gebremedhin et al., 2019). With advances in sensors, computing technologies, and more recently in artificial intelligence tools and methods, there has been a surge of interest around automated and high throughput phenotyping techniques for overcoming this bottleneck (Walter et al., 2012; Araus et al., 2018).

Numerous techniques have been developed over the last 15 years for assessing clover and ryegrass forage yield using image data. (Bonesmo et al., 2004) developed a method for pixel-level segmentation of clover and ryegrass using thresholding and morphological filtering on RGB images. This method was applied to predicting the visible area of clover within a specified region. They showed that their automated method could achieve correlation of R^2^ = 0.81 compared to marking out the same areas manually. This method was extended by (Himstedt et al., 2012) through performing segmentation in the HSV colour space. They then combined this information with a linear model for different legumes to estimate the legume biomass. Although high correlations between measured and predicted legume content were observed (R^2^ = 0.90–0.94), the validation set only consisted of 44 data points spread between their different legumes and monoculture examples. They also limited their validation method to swards with less than 2,800 kgDM/ha. (Mortensen et al., 2017) also investigated improvements to this method by looking at different ways of combining the colour information in RGB space. They were also able to obtain a biomass correlation of R^2^ = 0.93 with a multivariate linear model trained separately for mixed sward grown from different seed mixtures. This was done using a similar sized validation set, however spanning a larger forage dry matter range (up to 3,084 kgDM/ha). An issue all of these researchers found with the morphological approach is that the erosion operations have a tendency to eliminate small clover leaves producing a bias —especially in younger pasture. There are also significant differences between the camera setup, lighting, and image resolutions in each of their setups—requiring tuning of the parameters provided in (Bonesmo et al., 2004) and derivative methods to use them. (Skovsen et al., 2017) investigated use of deep learning for segmentation of clover and ryegrass. They trained the fully convolutional network for semantic segmentation (FCN) model (Shelhamer et al., 2014) using synthetic pasture data constructed from clover, ryegrass, and weed leaves cropped from photographs. The network was shown to have a significantly higher segmentation accuracy than the method in (Bonesmo et al., 2004)—with a pixel accuracy of 83.4% and mean intersection over union (IoU) of 65.5%. (Skovsen et al., 2017) did not regress their segmentation results directly to the clover dry matter as done by others mentioned. Instead their analysis was limited to the correlation of clover-vegetation fraction between dry matter measurements and estimated coverage area.

The use of deep learning in place of traditional machine vision techniques has become increasingly common since (Krizhevsky et al., 2012) demonstrated that CNNs are unreasonably effective for solving image classification type problems. Over the last five years, significant efforts have been made toward adapting CNNs to image segmentation. The majority of these networks follow the same general procedure: the image is first down-sampled while extracting semantic information, followed by up-samping and extrapolation of the semantic information back to the image's original size. The simplest segmentation network (FCN) makes coarse predictions from down-sampled features, then uses learned deconvolutions, skip-connections, and bilinear interpolation to upscale the predictions back to the original image size Shelhamer et al., 2016). SegNet built on this by following a more explicit encoder-decoder architecture, replacing deconvolutions with inverse-pooling operations that are subsequently followed by convolution layers (Badrinarayanan et al., 2016). The latest iteration of the DeepLab architecture (DeepLabV3+) is currently one of the more popular networks being used for high accuracy image segmentation. This replaces some of the down-sampling steps with separable atrous convolutions—which achieve the same effect without losing spatial resolution. They also reinforce the predictions using an additional decoder and global context information for the image (Chen et al., 2018). For a more general review on deep learning based image segmentation, we direct the reader to (Garcia-Garcia et al., 2017).

FCN was the first deep learning network architecture developed for pixel-level segmentation. In the two years between since its introduction and subsequent application to pasture segmentation there have been dozens of networks developed that can achieve significantly higher segmentation accuracy (Garcia-Garcia et al., 2017). State of the art networks such as DeepLabV3+, PSPNet, and EncNet all achieve an mean IoU above 80% on standard computer vision benchmark datasets (Zhao et al., 2017; Chen et al., 2018; Zhang et al., 2018). It is worth noting however that the benchmarks these networks are developed generally focus on urban settings—and therefore are not guaranteed to perform as well for agricultural applications. These benchmarks often contain images with objects sparsely distributed with relatively little occlusion. Pasture images however have objects densely clustered with a high degree of occlusion.

We reported on a mobile multisensor platform for high throughput phenotyping of ryegrass to augment selective breeding (Ghamkhar et al., 2018). Here, we build upon the work of (Skovsen et al., 2017) and (Ghamkhar et al., 2018), with the aim of improving the accuracy of clover/ryegrass segmentation. The focus of this paper is on the measurement of percentage white clover (*Trifolium repens* L.) and perennial ryegrass (*Lolium perenne* L.) yields in mixed sward using top-down view RGB images and deep learning. We introduce a new CNN architecture which we have named local context network (LC-Net) which applies design principles from the aforementioned networks to segmentation of complex agricultural images. We show that LC-Net can differentiate clover and ryegrass with a significantly higher accuracy than previously applied deep learning based methods reported by (Skovsen et al., 2017).

## Materials and Methods

### Data Collection

**Figure 1:**
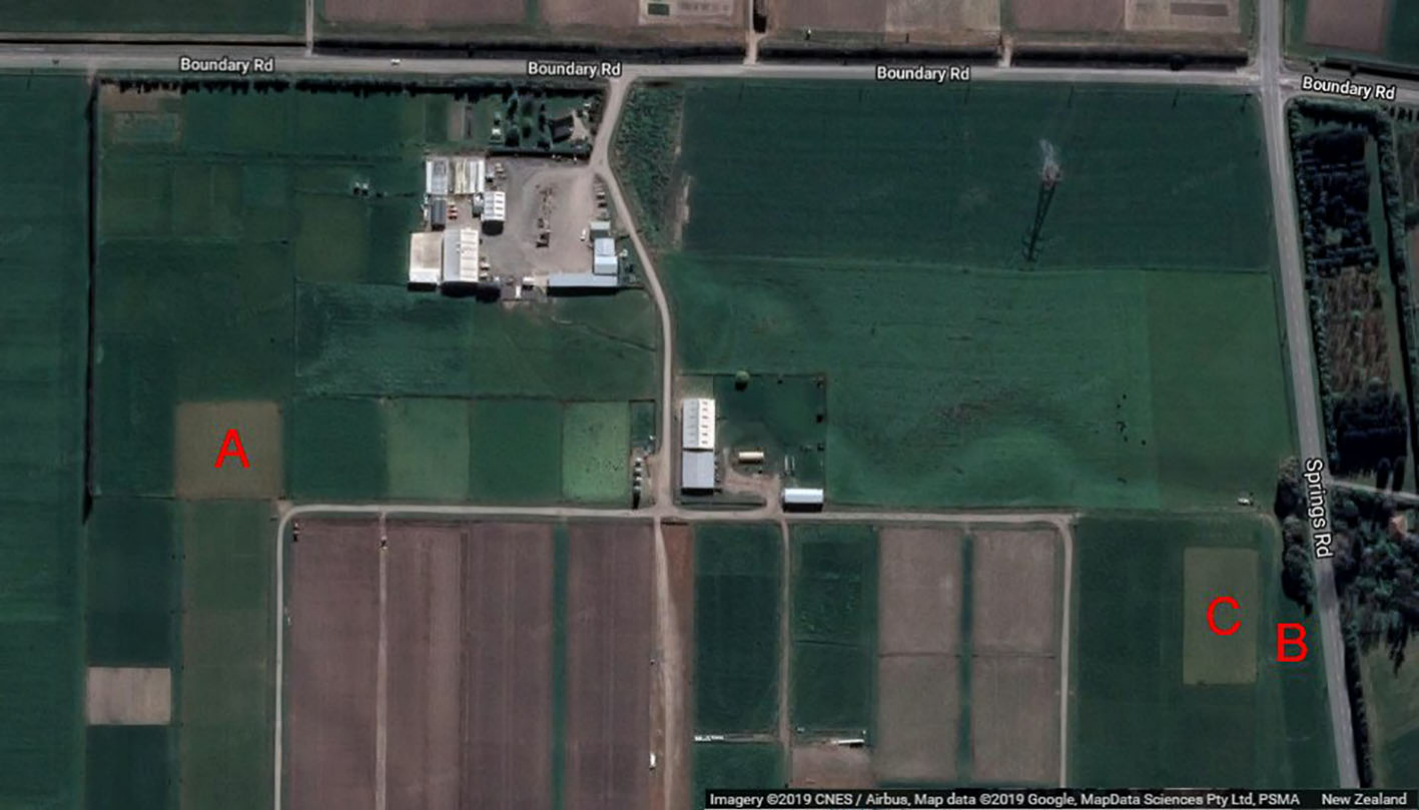
All data was collected at the AgResearch farm in Lincoln, New Zealand (Lat:-43.627799, Long:172.470908). Data used for training the neural network was collected from sites A and B. An independent mixed sward trial at site C was used for validating against harvest measurements. Satellite image retrieved from Google Maps (16 April 2019) and follows attribution guidelines for redistribution.

All data used in the manuscript was collected at the AgResearch Lincoln farm in Canterbury, New Zealand (−43.667799, 172.470908). Data used for training our neural network was gathered from location's A and B in **Figure 1**. A mixed sward trial at location C was used for independently comparing the RGB segmentation results to dry matter harvest measurements. All three of these locations contained varying mixtures of perennial ryegrass and white clover. Data was collected using our mobile data collection platform for forage assessment—the *Multiple Scanning Imaging Capacity System* (MSICS) described by (Barrett et al., 2018).

#### Mobile Data-Capture Platform

**Figure 2:**
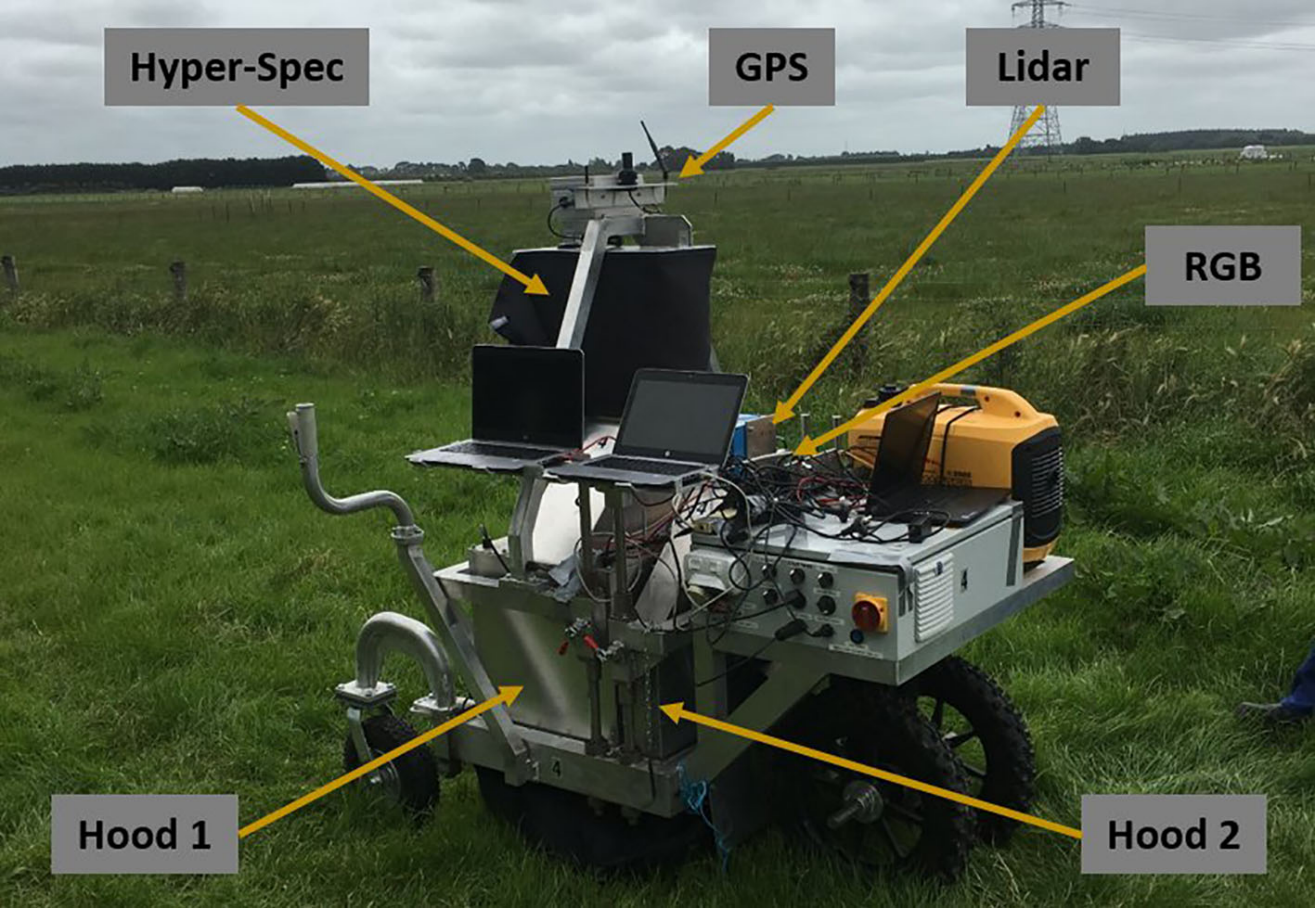
The multiple scanning imaging capacity system (MSICS) used for high throughput phenomic screening of forage. Hood 1 contains SWIR and VNIR hyperspectral cameras. Hood 2 contains light detection and ranging (LiDAR) and red, green, and blue (RGB) sensors. Each hood contains its own custom lighting setup. A black skirt is installed around the base of the hoods to block out ambient light. An encoder is installed on the back wheel for measuring distance. The MSICS also incorporates real-time kinematic global positioning system (RTK-GPS) technology to enable geo-referencing.

**Figure 3:**
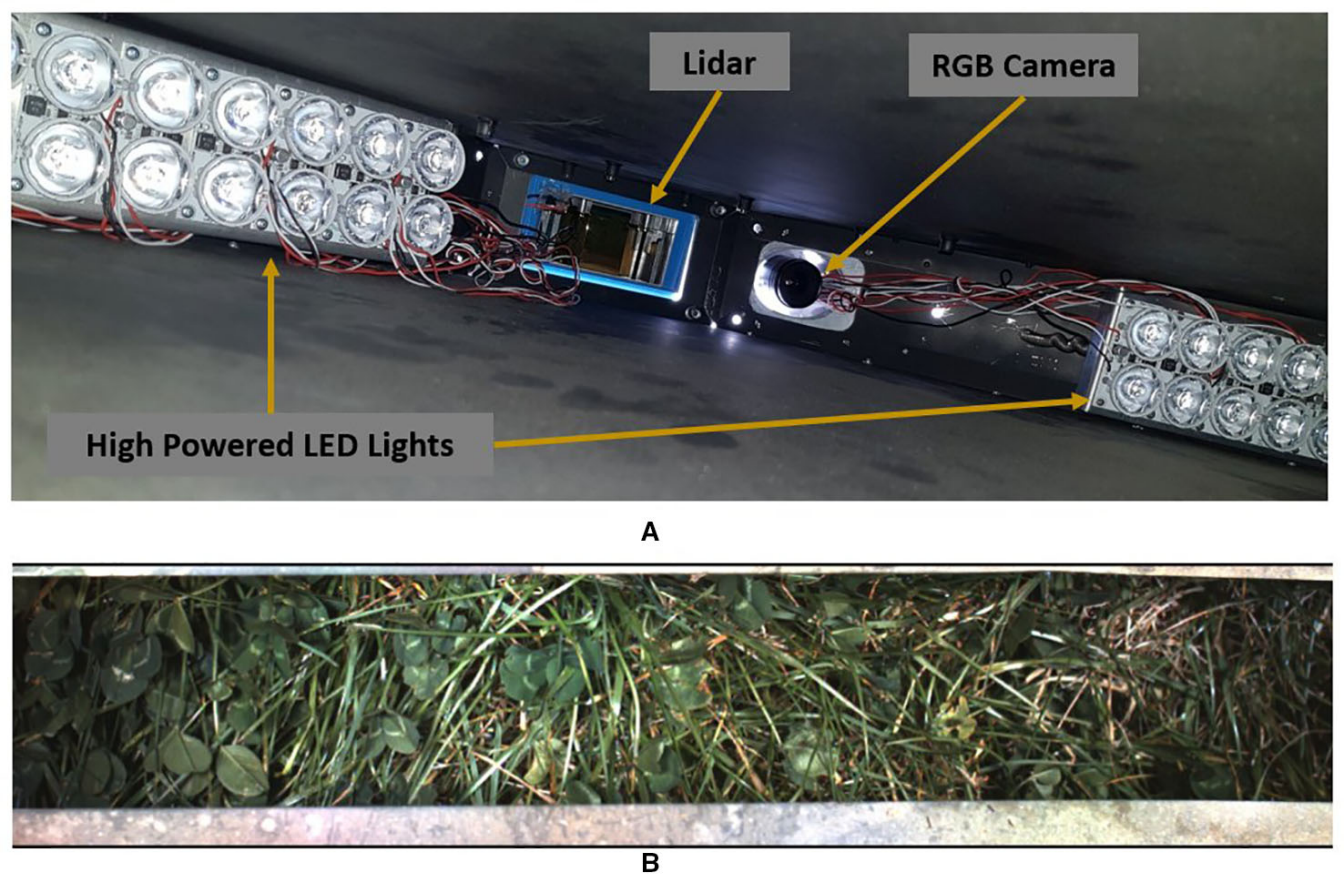
**(A)** Interior of Hood 2 on the multiple scanning imaging capacity system (MSICS) showing the custom lighting setup alongside the LiDAR an red, green, and blue (RGB) camera. **(B)** A typical image captured from the RGB camera during operation.

The MSICS contains a range of different sensors—including RGB, LiDAR, as well as VNIR and SWIR hyperspectral cameras, RTK-GPS, and a wheel encoder (**Figure 2**). The MSICS contains two hoods, each with controlled lighting specificly designed for the sensors mounted within the respective hood. The Teledyne Dalsa Genie Nano C1920 RGB camera used for this work is mounted in the second hood along with the LiDAR (**Figure 3A**). The underside of the hood is lined with a custom rig of focused high-powered RGB LED lights that illuminate the ground directly below the camera (**Figure 3B**). During these experiments the RGB camera was setup to acquire 14 frames per second. During data-capture the MSICS was driven at speeds ranging from 0.14 ms^–1^ to 0.37 ms^–1^.

#### Training Data

The data used for training our neural network was collected at location's A and B in **Figure 1**. RGB data was acquired while the MSICS was driven at full speed (approx. 0.4 ms^–1^) randomly across the mixed sward in each location. Note that the MSICS only had lighting installed on one side of the hood when data was collected at location B. The result of this is that approximately half of the data has deeper shadows than the other half. We retained this data in the final training set to improve the network's robustness to variable lighting conditions.

#### Validation Trial

A mixed sward trial at site C (**Figure 1**) containing ryegrass and clover was used for comparing visual clover fraction estimates to harvest measurements. The clover used in this trial are half white sib white clover (*Trifolium repens L.*) breeding lines along with two commercial checks, planted in October 2017. The grass cultivar is Rohan perennial ryegrass, which was sown in April 2017. Efforts have been made to minimise weed presence in the trial. Irrigation has not been used. The trial is row-column layout, with two replicates of 240 plots (a total of 480 plots). Each plot is approx 1.5 m by 1.5 m in size. The trial is scored when it reaches 2,800 kg/ha– 3,200 kg/ha (approximately 8–10 times per year), and then cattle grazed. The whole trial is scored for growth (total clover biomass) then a selection of plots, which are a fair representative of the whole trial are selected for harvest measurements. This equates to 20%–30% of the trial plots harvested, or approximately three representatives of each score per replicate. The trial was between 12 and 18 months old when the data for this paper was collected.

RGB data was collected for a subset of the harvested plots during two separate scoring events. First, a total of 30 measurements were taken on the 22*^nd^* January 2019, and then another 40 measurements were conducted on the 15*^th^* April 2019. Of these, five were taken at the edge of the trial to increase the amount of data collected with low clover fraction. Measured plots were chosen specifically to have a wide range of biomass and proportion of clover/ryegrass.

For each harvested plot, a 25 cm × 25 cm quadrat was placed on the plot in a position that visually corresponded to the plot's growth score (**Figure 6**). After RGB data was collected, the mixed material inside each quadrat was harvested using electric shears and packaged. Following this, clover and grass were separated from each sample before being dried and weighed.

### Training Dataset

To train convolutional neural networks, thousands of labelled images are typically needed. This problem is exacerbated for image segmentation where a label is required for every pixel. Although techniques for semisupervised and unsupervised learning do exist, their success varies between different problems. Therefore, we decided to construct our data set using two different labelling approaches. The majority of the data set is built from synthetic data that has been constructed using a variant of the synthetic image generation (SIG) method used by (Skovsen et al., 2017). Here samples of individual plants and leaves are cropped from RGB images captured by the MSICS, then randomly overlapped to create new images where the label is already known. Augmentations such as scaling, flips, and gamma transformations are also applied to these samples to further increase diversity of images in the dataset. This process enables the synthesis of thousands of unique labelled images from a relatively few number of samples. We have also included a number of partially labelled images to provide some examples that are more realistic and target configurations that are not easily simulated—such as clusters of small gaps in the pasture canopy, object boundaries, and complex shadowing effects. Each partially labelled image was augmented with vertical and horizontal flips to quadruple the amount of partially labelled data.

Our final training data set is made of 4,500 training images and 600 testing images as shown in **Table 1**. Samples used for generating synthetic data for the training and testing data sets were kept separate to avoid cross-contamination. Each image in this dataset have both a height and width of 100 pixels. We also experimented with larger image sizes, however the 100×100 size provided a good balance between training speed and image complexity.

**Table 1:** Composition of the training and testing data sets.

	Clover	Ryegrass	Background	Synthetic	Partial	Total
Training Set	308	230	54	2700	450 (x4)	4500
Testing Set	50	50	50	400	50 (x4)	600

Each set contained independent clover, ryegrass, and background samples. The amount of partially labelled data was quadrupled using flip augmentations.

### Synthetic Image Generation

**Figure 4:**
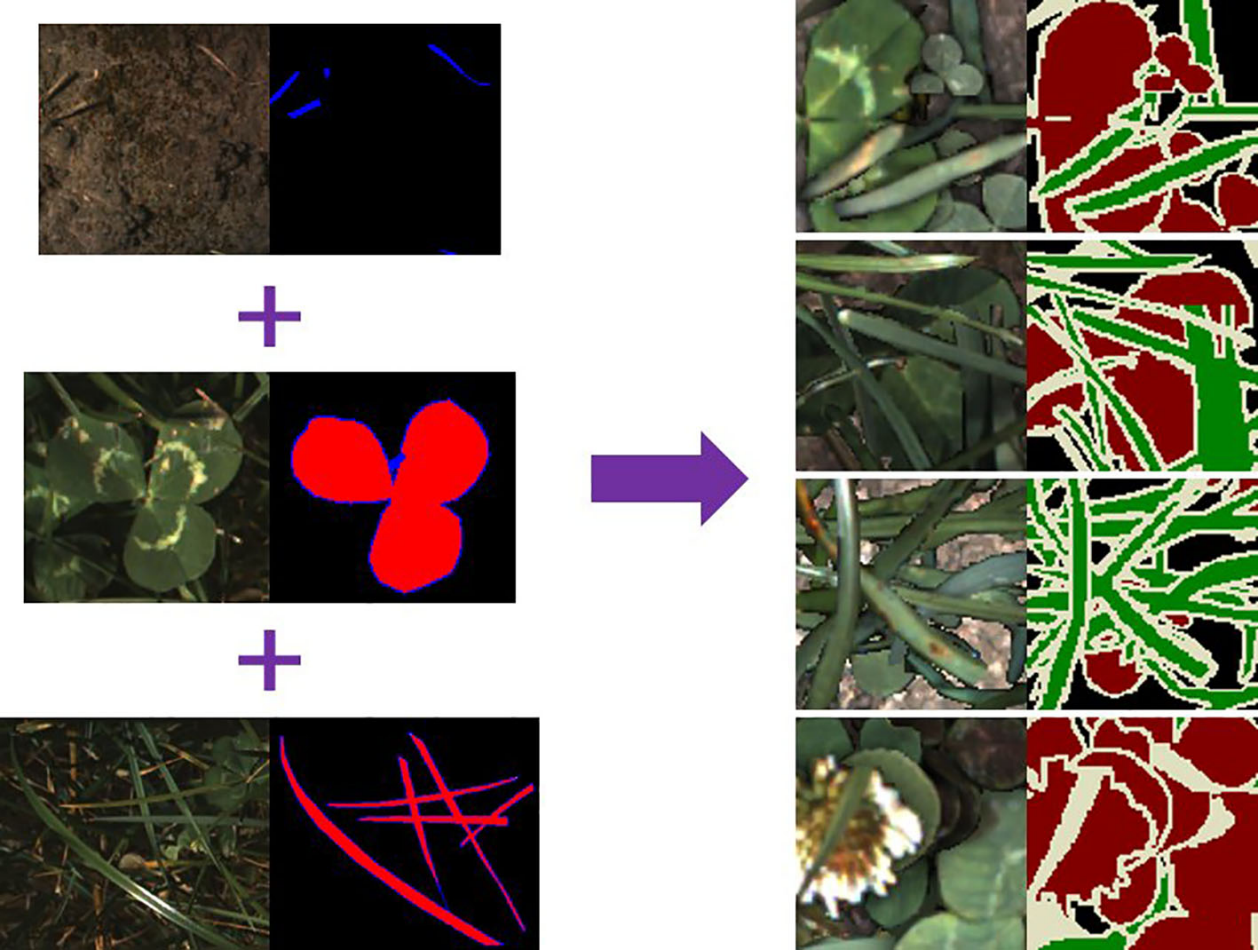
Example of synthetic image generation. Plant samples use the labelling convention: red = object of interest; blue = pixels to include but ignored during training—such as edges or ambiguous pixels; black = excluded from sample. The synthetically generated labels (right) use a convention based on the (Everingham et al., 2012) colour scheme: red = clover; green = grass; black = background; white = pixels marked to be ignored during training.

The SIG process we followed was similar to that used by (Skovsen et al., 2017). Each synthetic image contains a random background (soil image), and between 2 and 20 random samples that are augmented and overlaid in random positions. The selection probability for clover and grass samples was subjectively adjusted to account for the average sizes of each to ensure a balanced distribution of ratio and sparseness. Sample augmentations included: horizontal and vertical flips; scale ±25%; gamma ±10%; and saturation ±25%. The ground sample distance for our data is approximately 2–3 pixels per mm. As such we have omitted rotation augmentations of our samples as it disrupted the texture information at this resolution. We also apply Gaussian drop shadows to approximately half of our synthetic images to darken underlying soil and leaves. Instead of simulating shadows that are realistic for our data-capture system, we vary the extent and intensity of the Gaussian drop shadows in order to improve our networks robustness to different lighting conditions. Examples of our synthetic data are shown in **Figure 4**. Unlike Skovsen et al. who focused on making photo-realistic synthetic images, we have embraced the inelegance of simple stitching as a further form of data lighting augmentation. We also identify the boundary pixels in each sample so that they can be ignored during network training to reduce influence of edge artefacts.

### Local Context Network

**Figure 5:**
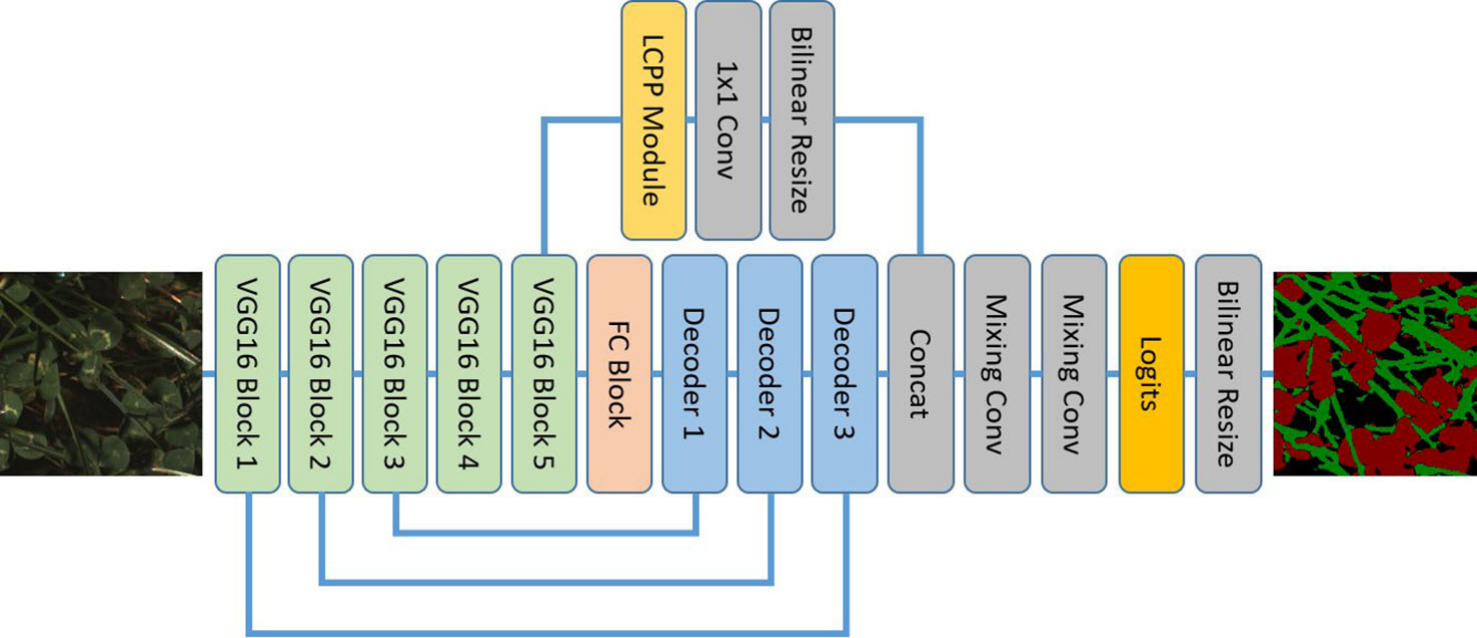
The local context network architecture using a VGG16 backbone and local context pyramid pooling.

LC-Net follows an encoder-decoder structure (**Figure 5**), taking design inspiration from a number of recent segmentation networks. The input image is encoded using the first five blocks of VGG16 (Simonyan and Zisserman, 2015). We have also followed standard practice of removing the max-pooling layer from final block. The decoder part of the network has two branches. The primary branch contains an FC block (equivalent role to the Fully Connected layers of VGG16) and three Decoder blocks which have skip connections to the corresponding encoding blocks. The second branch takes the features from the last VGG16 block and feeds them through a custom pyramid pooling module we have designed, which we have named local context pyramid pooling (LCPP). The output of the LCPP module is then resized to be the same size as the output of the primary branch (both in spatial and feature dimensions) and concatenated to it. This is then put through mixing convolution layers for combining the information from each branch before the logits are calculated. At this point the output has a stride of 2 and the up-sampling is completed with bilinear interpolation. Final predictions are then made by applying softmax activation to this output.

#### FC Block

This consists of three 3×3 convolutions then a 1x1 convolution. Each convolution is followed by ReLU activation then batch normalisation. We choose the number of output channels for these convolutions to be 1,024 as we generally find VGG16's original 4,096 feature length to be excessive for problems targeting a small number of classes.

#### Decoder Block

The output from the previous layer is bilinearly resized to match the output size of the corresponding VGG16 block output. These are then concatenated and passed through two mixing convolution layers. There are different numbers of channels for each input into the concatenation—this causes the less developed features in the earlier VGG16 blocks to have less influence on the semantic information in the final output.

#### Mixing Convolution

These follow the same design principle shown by the DeepLabV3+ decoder; i.e. it is beneficial to follow concatenation operations with several convolutions to properly combine the features. We use a 3×3 convolution followed by ReLU activation then batch normalisation for this. The number of output channels is fixed to 128 for all mixing convolutions in this network.

#### LCPP Module

The input to this module is sent separately through two average pooling layers with kernel sizes 3×3 and 5×5. “Same” padding is used to maintain spatial shape. The outputs of these pooling layers are then concatenated before being sent through two 1×1 convolution + ReLU activation + batch normalisation blocks. The length of the features returned by these convolutions are the same as the module input.

It has been demonstrated by other networks (Chen et al., 2018) that predictions can be improved by incorporating the global context vector (the image described by single rich feature vector with zero spatial extent) into the final decision process. However, it would be ambiguous to apply this with forage images of different sizes and scales (i.e., visually forage is relatively densely packed and continuous across the extent of the image). The LCPP module is designed to play the role of the global context vector in this situation. It does this by incorporating multiple context vectors that are local to large regions of the image at different scales. The dependencies on image size and scale are limited as a result.

### Network Training

LC-Net was trained to classify pixels to three classes—Clover, Ryegrass, and Background. The background class includes regions containing soil and exceptionally dark shadows. Pixels of other pastoral species are explicitly ignored during training—therefore will have indeterminate classification during inference. ImageNet initialisation was used for the VGG16 layers (Tensorflow, 2016). The other weights were initialised using standard Xavier initialisation. The network was trained for 400 epochs with a batch size of 24, using Adam optimisation, partial categorical cross-entropy loss and a learning rate of 5e-5. Training LC-Net with the above data set took 3.5 h on a desktop PC using a GTX 1080Ti GPU and an Intel i7-7700K CPU.

### Data Postprocessing

Segmentation networks provide a prediction score for each class in every pixel which is used to determine the classification for that pixel. After processing each image with the network, we defined any pixel with a prediction confidence less than 80% to be background (i.e., uncertain predictions). All other pixels were defined as as either clover or ryegrass depending on which the had the highest prediction confidence for the respective pixel.

When processing the data for the validation trial the harvest frame did not fit in the camera field of view in the driving direction. Furthermore, the height of the hood was adjustable—meaning that the camera footprint (i.e., pixels/mm) needed to be calculated dynamically. A rasterising-like approach to stitching the RGB data was taken as per below.

**Figure 6:**
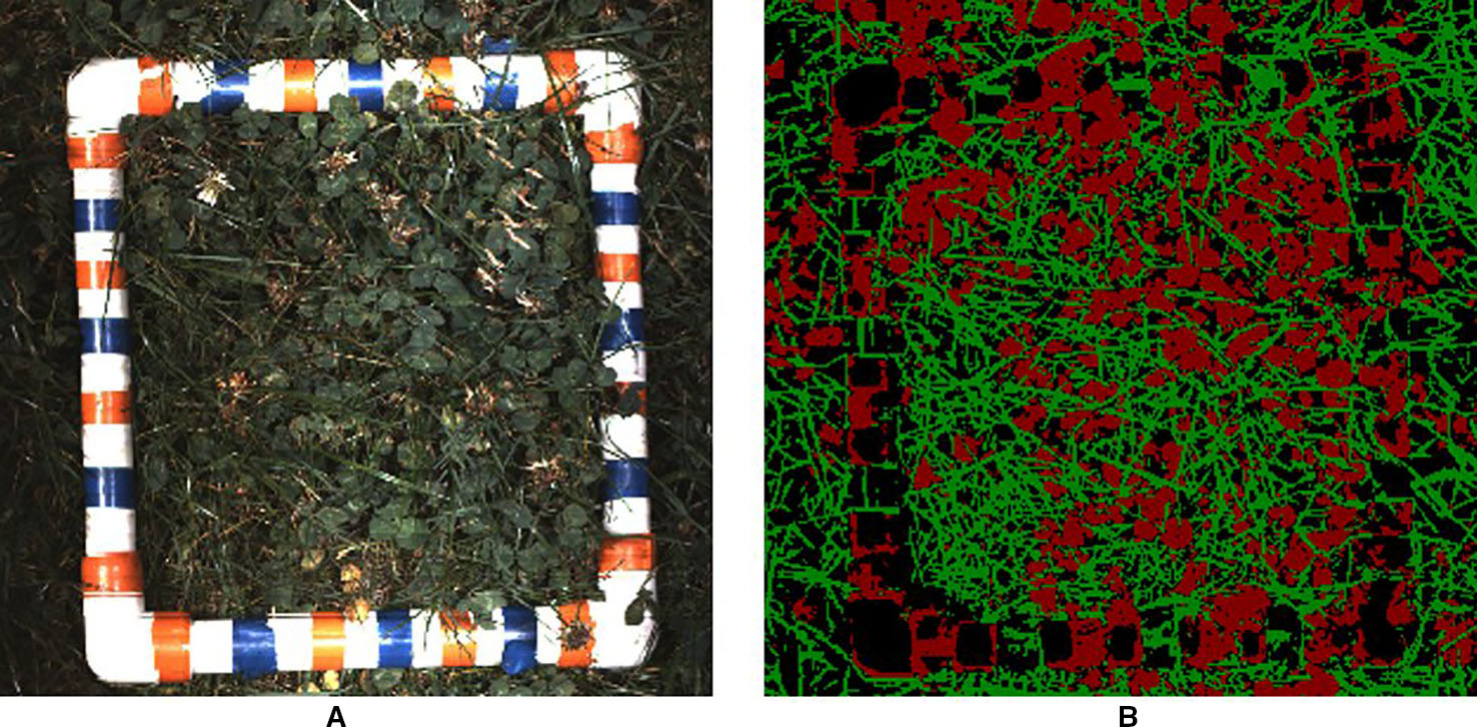
**(A)** visual and **(B)** segmentation images for one of the validation trial plots after rasterising. Each image has a pixel size of 1.2 × 1.2 mm^2^. The harvest frame has ambiguous segmentation as the convolutional neural network (CNN) has not been trained to recognise it.

First cross-correlation between adjacent images was performed to determine the number of overlapped pixels. The overlap strip was then divided into 22 strips, taking the central row of pixels as the representative for each strip. This was then followed by using the wheel encoder information to determine the distance travelled in this overlap region, and therefore the relative position of each strip. The pixels/mm along each strip is determined from information obtained by the LiDAR unit on the MSICS. These samples were then averaged in a raster grid with a pixel size of 1.2 × 1.2 mm. An example of this rasterising process applied to both an RGB image and its associated segmentation mask is shown in **Figure 6**. The rasterised RGB image was not processed with LC-Net. Instead, LC-Net is used to create segmentation masks for the raw RGB images. The same rasterising parameters calculated for stitching the raw RGB images is also used to stitch the associated segmentation masks.

## Results

### Network Training

**Figure 7:**
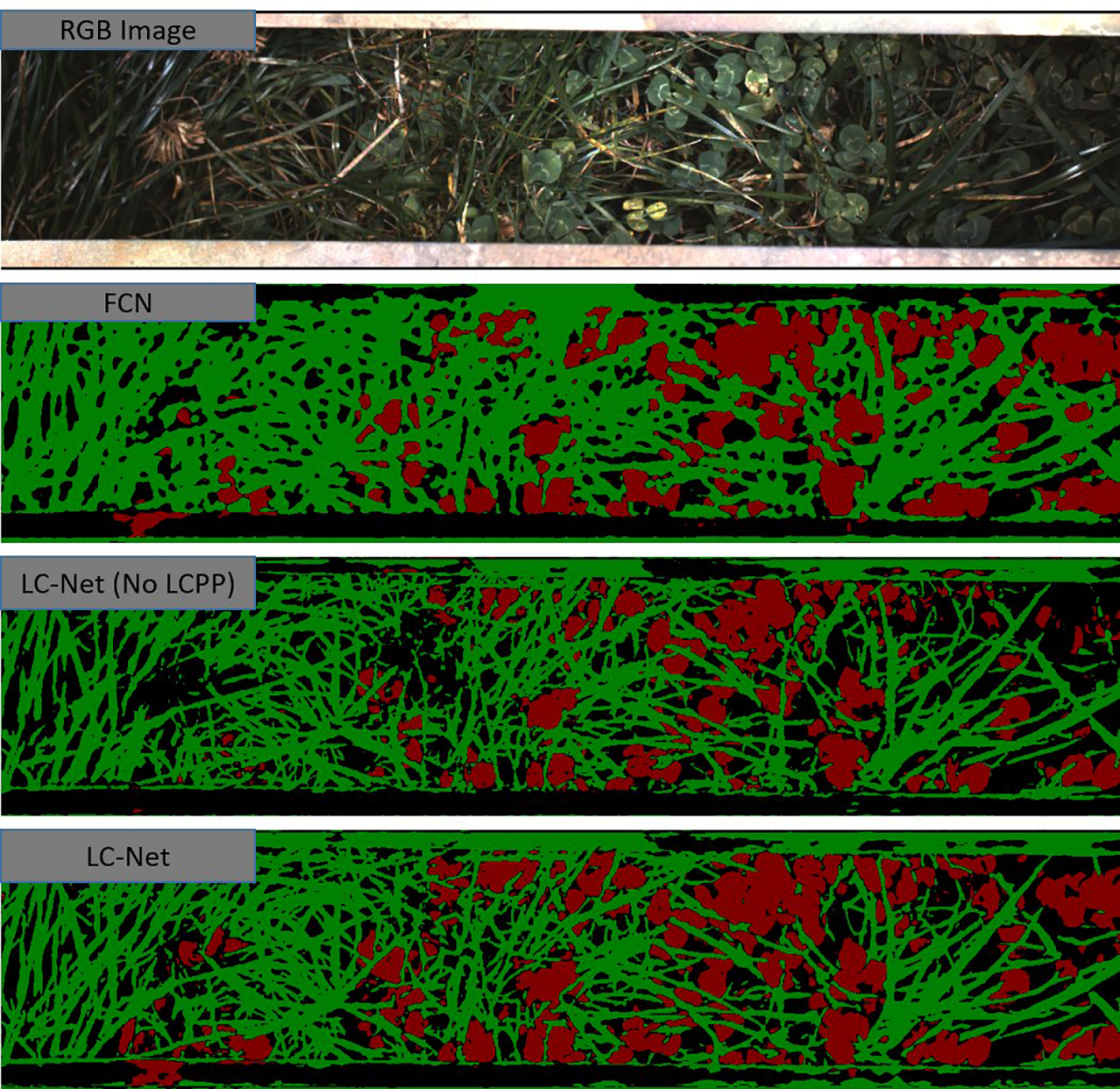
A red, green, and blue (RGB) image that has been captured by the multiple scanning imaging capacity system (MSICS) at site C, and processed using each of the trained networks—fully convolutional network for semantic segmentation (FCN), local context network (LC-Net) [without the local context pyramid pooling (LCPP) module], and the full LC-Net. The segmentation masks show clover (red), grass (green), and background (black). The background class includes soil, very dark shadows, and pixels that have less than an 80% confidence score in their classification.

In addition to LC-Net, two more networks were trained for comparison (**Table 2** and **Figure 7**). We used two standard metrics to assess the quality of each network—mean pixel accuracy and mean IoU. Both of these metrics are class weighted averages. Due to the non-linear behaviour of these metrics the mean IoU was the more informative one during training after the pixel accuracy exceeded 90%.

**Table 2:** Comparison of networks trained for clover-ryegrass segmentation.

Method	mAcc	mIoU	mAcc (CV)	mIoU (CV)
FCN (Skovsen et al., 2017)	83.4	65.5	–	–
FCN	92.7	74.8	92.0	73.4
LC-Net (without LCCP)	93.7	79.1	93.4	77.3
LC-Net	**95.4**	**81.3**	**95.0**	**79.0**

The mean pixel accuracy (mAcc) and mean intersection over union (mIoU) metrics are class-weighted averages. The first two columns are results for our original dataset configuration. The last two columns are averaged results from a stratified fivefold cross-validation (CV).

FCN was trained for one of the comparison networks. Although data from (Skovsen et al., 2017) consists of higher resolution images, the FCN model we trained in our study significantly higher accuracy—with an IoU close to that reported for benchmark datasets (Shelhamer et al., 2016). As illustrated in **Figure 7**, FCN lacks the ability to properly delineate fine filament like structures or corners, which is fundamental limitation of this architecture.

To show the benefits provided by the LCPP module, the LC-Net architecture both with and without the LCPP branch. Despite the LCPP module only improving the mean IoU by 2.2%, there is a significant boost in performance as presented in **Figure 7**. Clover in the segmentation are more filled in; and prominent gaps in the grass from the network without LCPP are filled in when LCPP is included. Overall, the full LC-Net outperformed all other networks tested for this application.

**Figure 8:**
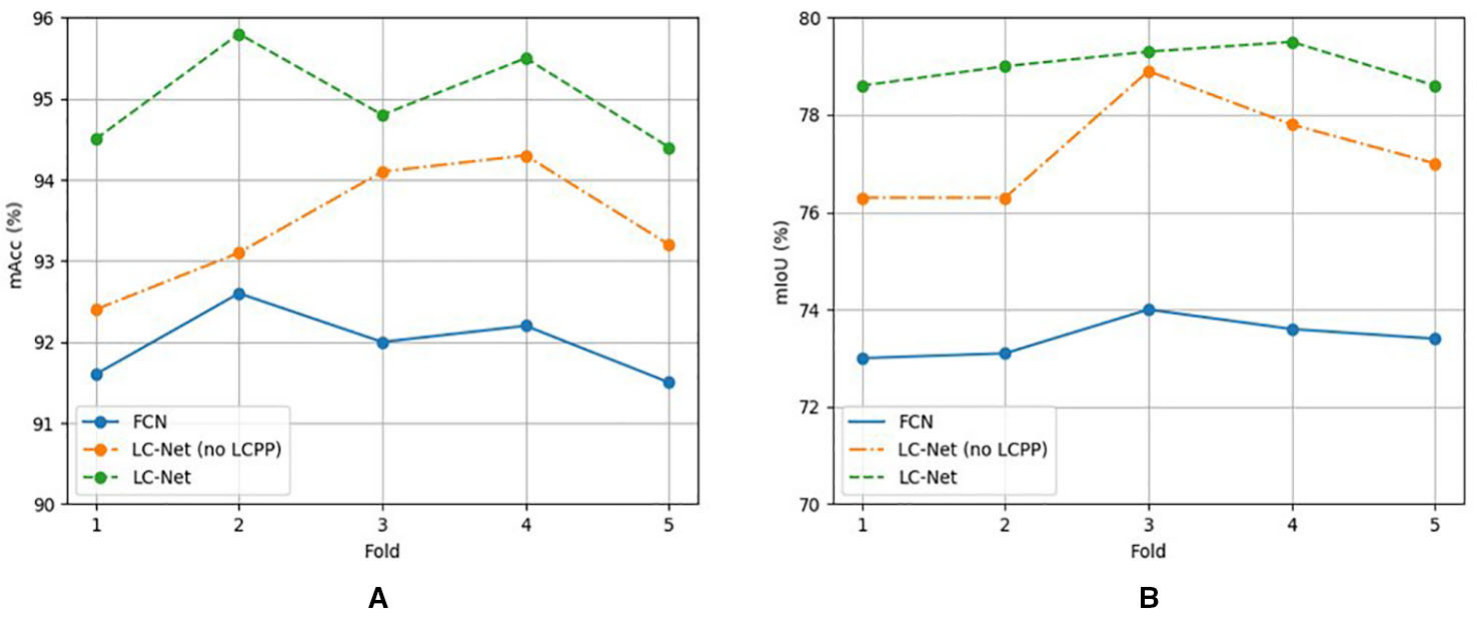
Mean accuracy **(A)** and mean intersection over union **(B)** results for each fold for models trained using stratified fivefold cross-validation. Results are relatively consistent between folds for each model.

To test the robustness of our training process and dataset we applied a stratified 5-fold cross validation to each of the tested networks (**Figure 8**). We split each sub dataset (17/07/2018 samples, 05/12/2018 samples, and partially labelled) independently and aggregated the respective folds. This was so the samples with different lighting conditions would not be mixed in the synthetic data generation process. The accuracy and mIoU for these cross validation runs are on average a few percent lower than obtained from the original dataset configuration (**Table 2**). However, the overall network ranking remains unchanged. Note the original dataset configuration used approximately 11% more samples in the training set than the cross validation. This indicates that further improvements may still be gained through increasing the number of samples in the dataset.

### Sward Composition

To assess the improvement in estimating sward composition using LC-Net over FCN we used 70 data points from our validation trial. A small number of data points were excluded from this analysis due to insufficient data for to completing the rasterising process. The fraction of clover estimated from the RGB images were taken as the ratio of clover pixels to clover and ryegrass pixels as identified by the respective networks. The clover fraction for the harvest measurements is obtained from the equivalent calculation using dry weights instead of pixels.

**Figure 9:**
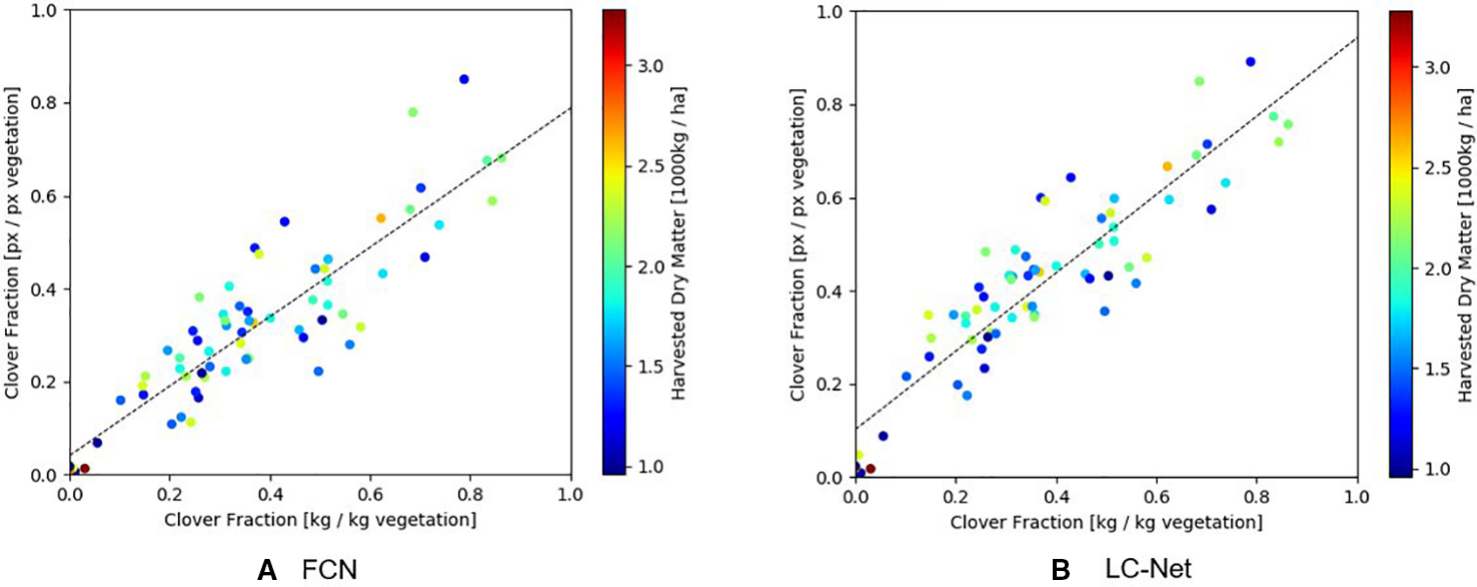
Comparison of the clover-vegetation ratio obtained from RGB images and harvested dry matter measurements. **(A)** Red, green, and blue (RGB) results from the fully convolutional network for semantic segmentation (FCN) network has a linear correlation of R^2^ = 0.793 with harvested dry matter. **(B)** RGB results from the local context network (LC-Net) and has a linear correlation of R^2^ = 0.825 with harvested dry matter.

As shown in **Figure 9**, the clover fraction correlation obtained from LC-Net (R^2^ = 0.825) is only marginally better than what was obtained for FCN (R^2^ = 0.793). Overall this similarity is not surprising since they provide approximations to the same coverage areas. Data points with similar forage density are also spread relatively evenly throughout the scatter cloud, which suggests that a significant component of the variation is due to occlusion of underlying vegetation by the top layer of pasture.

## Discussion

Deep Learning methods have a tendency to produce results that appear much better compared to traditional methods, especially on classification type problems. However, since the field of deep learning is advancing at a very high rate it is challenging for other fields to uptake state-of-the-art techniques into new applications. The first pre-print of the FCN network was published two years before it was applied to segmenting ryegrass from clover (Shelhamer et al., 2014; Skovsen et al., 2017). During this time dozens of new segmentation networks were published that significantly outperformed FCN (Garcia-Garcia et al., 2017). Also, most CNN architectures have been developed on standard computer vision benchmark data sets, many of which are focused on urban settings. Agricultural applications can offer challenges that are either not present or less prominent than those found in these benchmarks. For example, the object detection networks such as Faster R-CNN and YOLO are now commonly applied to fruit and weed detection. However, these networks have severe limitations around how many objects they can detect which can cause problems when looking at a tree with hundreds of targets—requiring them to be applied in innovative ways. Based on our experience in this research, global context information for proximal pasture images contain sufficient ambiguity to make it challenging to robustly incorporate it into networks for this application. During the development of LC-Net we trialled DeepLabV3+ (a state-of-the-art network which utilises global context) on this problem. We found that it required both a fixed ground sample distance and a consistent input image size between both the training and deployment versions of the model. This restriction is not practical as it is too time consuming and costly to train and maintain a different model for every potential hardware configuration we may need to use. The LCPP module is a pragmatic alternative to global context—providing similar benefit without being restrictive on the development of our data-collection platform.

For this work we did not restricted ourselves to using predefined CNN architectures, instead we developed our own specifically for the application of canopy segmentation in an agricultural context. In this paper we have applied used this new architecture (LC-Net) for analysing pasture. The design of LC-Net is influenced by a number of different networks. Although we don't use separable convolutions, the decoder modules and mixing convolutions are derived from the structures used for DeepLabV3+. The network also trained more robustly when we used skip-connections during up-sampling rather than atrous convolutions—although it is not clear whether this observation is specific to our application. We also used a pyramid pooling module of our own design (LCPP) to incorporate local context information at different scales instead using a single global context. By including a context layer in our network, we effectively force it to make a high level visual score and incorporate that into the decision process. Another advantage of LCPP is that it uses pre-defined pooling operations, therefore requires significantly fewer trainable parameters than other pyramid pooling techniques. The description for LCPP we have given is very specific, however it follows a generic formula. It can be adapted by changing the number of pooling layers along with different kernel sizes and/or changing the number of convolutions and feature sizes following the concatenation. The design of this module will almost certainly need tuning for specific applications/data depending on factors such as relative object size. This aspect is being investigated further. Our experience working with CNNs is that those built upon VGG16 are easier to train for canopy assessment compared to deeper networks such as Xception (Chollet, 2017) and ResNet (Kaiming et al., 2016). This is the primary reason for choosing VGG16 as the backbone of the first iteration of our LC-Net. In addition to pasture, we are also currently investigating extensions of LC-Net for segmentation of orchard and vineyard canopy applications, in addition to how well it performs on the standard benchmark data sets.

We showed that LC-Net significantly out-performs FCN for segmenting clover from ryegrass in mixed swards. Comparing our FCN results and those of (Skovsen et al., 2017) two major differences are noticeable. First, our images have half the ground sample distance of theirs. Given that FCN has an output stride of 8, the edges of objects in its predictions should be more uncertain with our data. Second, (Skovsen et al., 2017) included a class for predicting weeds whereas we have not. They used very few examples of weeds in their training data which negatively impacted their reported accuracy. It is likely their FCN model would have an accuracy similar to ours if either more weed examples were added to their data set—or if the weed was class removed entirely. Over the following season we are planning to incorporate other plants into our dataset so we can also adapt our network to be effective in weedy conditions.

We are planning to publish the dataset associated with this work separately. Before doing so however, there are several additions and improvements that we believe would add significant value—which include labelling data from different seasons, and inclusion of additional plants/weeds. It is worth noting that the dataset we have compiled for this study is highly specific to the camera and lighting setup we have used. For example, a few minor tests we performed demonstrated that our network is not generalised for natural lighting conditions (a drop in mIoU of roughly 30 − 40%). As such, networks trained by our dataset will likely perform poorly on data collected from setups substantially different from our own.

Comparing LC-Net and FCN models by the correlation between visual and harvested clover ratios, we see little difference in the results. Work by (McRoberts et al., 2016) and (Rayburn, 2014) using more traditional methods showed that it is also possible to obtain reasonable estimation through classification with coarse super-pixels and sparse subsampling. This indicates that significant boosts in pixel-level segmentation accuracy only translates to small (sometimes negligible) improvements in clover-vegetation fraction estimation with RGB imaging. There are two situations we identify that could benefit from higher segmentation accuracy: (1) when combining visual information with that of other sensors such as LiDAR to which can potentially compensate for the lack of plant density, volume, and occlusion information; and (2) when assessing pasture for more species than just clover and ryegrass. We are currently investigating both of these areas. Another motivation for using deep learning models is that they are in many cases simpler to work with, maintain, and extend when compared to more traditional feature crafting methods.

An observation of significance we made during training each of these networks was that training and testing losses could not be used to monitor for overfitting. After approximately 20–30 epochs in every training run the testing loss starts to increase (a typical sign of the network memorising the training set), however the accuracy and IoU metrics continued to improve regularly. This increase in validation loss seemed to be focused around the edges of the leaves in the images. Due to the mesh-like structure of pasture there is an unusually high proportion of class boundary pixels to interior pixels (%56 in our training data) that therefore provide a significant contribution to the loss. The synthetic images in our data set has been constructed in a manner that provides little information to the network about what true edges look like. This peculiarity may be resolved through improvements to how the training set is labelled, however the cost is likely higher than the benefit from doing so. The networks trained appear to work well in practice despite the elevated loss, and the accuracy metrics did not indicate overfitting nor appear to be significantly affected by this.

Overall, our results for assessing clover fraction using convolutional neural networks are comparable to those obtained by (Skovsen et al., 2017). We have demonstrated that relationship between visual and harvested clover fraction is reproducible using different networks, lighting setup, camera, image resolution, less photogenic synthetic data, and different postprocessing procedures.

## Conclusions

A new CNN architecture (LC-Net) designed for segmentation of agricultural canopies is showing promise for component identification in mixed sward. This architecture can segment clover and ryegrass in RGB images with higher accuracy than any other methods publicly available for the same application. We have also achieved this with half of the image resolution (pixels/mm) used by the next best method. Our comparisons between visual and harvested dry matter clover-vegetation ratios indicate that these improvements in segmentation accuracy do not yield similar improvements to biomass estimation. However, we predict that refined segmentation is necessary for improving biomass predictions when it is combined with information from other sensors.

## Data Availability Statement

The raw data supporting the conclusions of this article will be made available by the authors, without undue reservation, to any qualified researcher.

## Author Contributions

CB was the primary developer of LC-Net along with writing the associated code, performing the analysis, and writing the paper. JF and JH aided in the development of LC-Net through discussions around critical design choices and software debugging. JH also contributed to the literature review. Data collection and preprocessing was performed by CB, KI, AHe, and AHi. KI and MH developed the RGB image capture software used, the LED rig, and configured the setup. BJ installed the RGB hardware into the MSICS and was involved in the integration design decisions. SG oversaw and managed MSICS hardware changes and developments. KG guided the style of the manuscript writing and managed and directed the overall programme of research. All authors contributed to review and editing of the paper.

## Funding

This work was supported by Pastoral Genomics, a joint venture co-funded by DairyNZ, Beef+Lamb New Zealand, Dairy Australia, AgResearch Ltd, New Zealand Agriseeds Ltd, Grasslands Innovation Ltd, and the Ministry of Business, Innovation and Employment (New Zealand).

## Conflict of Interest

The authors declare that this study received funding from Pastoral Genomics—a joint venture co-funded by DairyNZ, Beef+Lamb New Zealand, Dairy Australia, AgResearch Ltd, New Zealand Agriseeds Ltd, Grasslands Innovation Ltd, and the Ministry of Business, Innovation and Employment (New Zealand). Pastoral Genomics has interests in developing technology that has potential to be commercialised for adding value to the forage breeding industry, and have involvement in setting the overall goals and vision of the Pastoral Genomics research programme. This relationship did not influence study design, data collection and analysis, decision to publish, or preparation of the manuscript. Lincoln Agritech Ltd. and Red Fern Solutions are independent research organisations that were subcontracted to conduct part of this research. Development of the LC-Net architecture was internally funded by Lincoln Agritech Limited. All other authors declare no competing interests.

The handling editor is currently organizing a Research Topic with one of the authors KG and confirms the absence of any other collaboration.

## Abbreviations

CNN, convolutional neural network; DM, dry matter; FCN, fully convolutional network for semantic segmentation (Shelhamer et al., 2016); HSV, hue, saturation, value; IoU, intersection over union; LC-Net, local context network; LCPP, local context pyramid pooling; LED, light emitting diode; LiDAR, light detection and ranging; MSICS, multiple scanning imaging capacity system; RGB, red, green, and blue; RTK-GPS, real-time kinematic global positioning system; SIG, synthetic image generation.`
